# Bear Canyon Virus: An Arenavirus Naturally Associated with the California Mouse (*Peromyscus californicus*)

**DOI:** 10.3201/eid0807.010281

**Published:** 2002-07

**Authors:** Charles F. Fulhorst, Stephen G. Bennett, Mary L. Milazzo, Hugh L. Murray, James P. Webb, Maria N.B. Cajimat, Robert D. Bradley

**Affiliations:** * University of Texas Medical Branch, Galveston, Texas, USA; † Orange County Vector Control District, Garden Grove, California, USA; ‡ County of Riverside, Department of Environmental Health, Hemet, California, USA; § Texas Tech University, Lubbock, Texas, USA

**Keywords:** Bear Canyon virus, *Arenaviridae*, Tacaribe serocomplex, *Peromyscus californicus*, California mouse

## Abstract

Thirty-four rodents captured in southern California were studied to increase our knowledge of the arenaviruses indigenous to the western United States. An infectious arenavirus was isolated from 5 of 27 California mice but none of the 7 other rodents. Analyses of viral nucleocapsid protein gene sequence data indicated that the isolates from the California mice are strains of a novel Tacaribe serocomplex virus (proposed name “Bear Canyon”) that is phylogenetically most closely related to Whitewater Arroyo and Tamiami viruses, the only other Tacaribe serocomplex viruses known to occur in North America. The discovery of Bear Canyon virus is the first unequivocal evidence that the virus family *Arenaviridae* is naturally associated with the rodent genus *Peromyscus* and that a Tacaribe serocomplex virus occurs in California.

The virus family *Arenaviridae* comprises two serocomplexes (1). The lymphocytic choriomeningitis-Lassa (Old World) complex includes lymphocytic choriomeningitis (LCMV), Lassa (LASV), Mobala, Mopeia, and Ippy viruses. The Tacaribe (New World) complex includes Whitewater Arroyo virus (WWAV), Tamiami (TAMV), Allpahuayo (ALLV), Flexal (FLEV), Paraná (PARV), Pichinde (PICV), Pirital (PIRV), Amapari (AMAV), Guanarito (GTOV), Junin (JUNV), Machupo (MACV), Sabiá (SABV), Tacaribe (TCRV), Oliveros (OLVV)**,** and Latino (LATV) viruses. Heretofore the only arenaviruses known to occur in North America were LCMV, WWAV, and TAMV.

Six arenaviruses are known to cause severe disease in humans (1). LCMV is an agent of acute central nervous system disease and congenital malformations; LASV is the agent of Lassa fever in western Africa; and JUNV, MACV, GTOV, and SABV are etiologic agents of hemorrhagic fever in Argentina, Bolivia, Venezuela, and Brazil, respectively. The occurrence of human disease caused by these viruses ranges from sporadic to hyperendemic.

Specific rodents are the principal hosts of the arenaviruses for which natural host relationships have been well characterized (2). The ubiquitous house mouse (*Mus musculus*) is the principal host of LCMV, woodrats (*Neotoma* species) in the southwestern United States are the principal hosts of WWAV, and the hispid cotton rat (*Sigmodon hispidus*) in southern Florida is the principal host of TAMV (2–8).

Antibody to an arenavirus recently was found in California mice (*Peromyscus californicus*) captured in the Santa Ana Mountains in Orange County, California (9). The purpose of our study was to determine the identity of the arenavirus associated with such mice in southern California.

## Methods

Rodents were captured at three sites near the Riverside-Orange County line: the Bear Canyon Trailhead (Riverside County: 33°36.7´N, 117°25.5´W), “El Cariso #1” (Orange County: 33°39.8´N, 117°25.1´W), and “El Cariso #2” (Orange County: 33°39.8´N, 117°25.7´W). The rodent fauna in the study area included the dusky-footed woodrat (*Neotoma fuscipes*), desert woodrat (*N. lepida*), brush mouse (*P. boylii*), California mouse, cactus mouse (*P. eremicus*), deer mouse (*P. maniculatus*), western harvest mouse (*Reithrodontomys megalotis*), California pocket mouse (*Chaetodipus californicus*), agile kangaroo rat (*Dipodomys agilis*), California ground squirrel (*Spermophilus beecheyi*), and Botta’s pocket gopher (*Thomomys bottae*) (S.G. Bennett, unpub. data).

Rodents were captured in Sherman traps (H.B. Sherman Traps, Inc., Tallahassee, FL) or Tomahawk live traps (Tomahawk Live Trap Co., Tomahawk, WI). Fifty traps were set at the Bear Canyon Trailhead on November 12, 1998; 20 traps were set at both El Cariso #1 and El Cariso #2 on June 10, 1998. A blood sample was collected from each rodent, and the blood samples and rodent carcasses were shipped on dry ice to the University of Texas Medical Branch. Subsequently, the carcasses and samples of lung, heart, liver, and skeletal muscle were deposited in the Museum of Texas Tech University, and mammalogists at Texas Tech University identified each rodent to species level on the basis of morphologic features of the animal’s skin and skull.

The blood samples were tested for antibody to WWAV by using an enzyme-linked immunosorbent assay (9). The test antigen was a detergent extract of Vero E6 cell monolayers infected with the WWAV prototype strain AV 9310135. The control (comparison) antigen was prepared from uninfected Vero E6 cell monolayers in a manner quantitatively identical to that used to prepare the test antigen. Serial fourfold dilutions (from 1:80 through 1:5,120) of each blood sample were tested against both antigens. The adjusted optical density (AOD) of a blood-antigen reaction was the OD of the well coated with the test antigen less the OD of the corresponding well coated with the control (comparison) antigen. A sample was considered to be antibody-positive if the AOD at 1:80 and 1:320 both were >0.200 and the sum of the AODs for the series of fourfold dilutions (from 1:80 through 1:5,120) was >0.750. The criteria for antibody positivity were based on the results of a laboratory study of white-throated woodrats (*N. albigula*) experimentally infected with the WWAV prototype strain AV 9310135 (10). The antibody titer of a positive sample was the reciprocal of the highest dilution of that sample for which the AOD was >0.200.

A 10% wt/vol crude homogenate of brain tissue from each animal was tested for infectious arenavirus by cultivation in a monolayer of Vero E6 cells (4). Cells scraped from the monolayer on day 13 after inoculation were tested for arenaviral antigen by an indirect fluorescent antibody test. The primary antibody in that test was a hyperimmune mouse ascitic fluid prepared against the WWAV prototype strain AV 9310135.

The nucleotide sequence of a 489-nt fragment of the nucleocapsid protein (NP) gene of isolate A0070039 and the sequence of the homologous fragment of the NP gene of each of the four other viral isolates from rodents included in this study were determined (Table). Subsequently, the nucleotide sequence of a 616-nt fragment of the NP gene of isolate A0070039 was determined. The 616-nt fragment included the entire 489-nt NP gene fragment and represented the genomic region that has been the basis of several comprehensive studies on the phylogeny of the arenaviruses (11–15). Total RNA was extracted from infected Vero E6 cells by using TRIzol Reagent (Life Technologies, Inc., Rockville, MD). Reverse transcription (RT) and polymerase chain reaction (PCR) amplification of the 489-nt gene fragment were performed with the Access RT-PCR Kit (Promega Corp., Madison, WI) in conjunction with oligonucleotides AVNP3 (5´-TCTTGATGACTATTCCCTTATGC-3´) and AVNP4 (5´-AACACTGTGGTTGAGTTTGATAG-3´). RT-PCR amplification of the 616-nt gene fragment was done by using SuperScript II Rnase H^-^ Reverse Transcriptase (Invitrogen Life Technologies, Carlsbad, CA) and PCR SuperMix (Invitrogen Life Technologies) in conjunction with oligonucleotides ARE3´-END, and AVNP7 (5´-TCTGGAGAAGGATGGCC-3´) and AVNP8 (5´-ACATGATACAATCCATCAATGCACAGTG-3´), respectively (16). PCR products of the expected size (535 bp or 661 bp) were purified from agarose gel slices with the QIAquick Gel Extraction Kit (Qiagen, Inc., Valencia, CA). Both strands of each purified PCR product were sequenced directly by using the dye termination cycle sequencing technique (Applied Biosystems, Inc., Foster City, CA) in conjunction with oligonucleotides AVNP3 and AVNP4 or AVNP7 and AVNP8. The nucleotide sequences generated in this study were deposited in the GenBank nucleotide sequence database under accession nos. AY093616 and AF497572 through AF497575.

**Table:** Results of tests for arenaviral infection in 10 antibody-positive or virus-positive rodents, California

Rodent	Species	Date captured	Trap site^a^	Antibody titer^b^	Viral strain
TK 90425	*Neotoma fuscipes*	11/13/98	BCN	>5,120	—
TK 90430	*Peromyscus californicus*	11/13/98	BCN	>5,120	—
TK 90435	*P. californicus*	11/13/98	BCN	>5,120	—
TK 90438	*P. californicus*	11/13/98	BCN	1,280	AV A0070039
TK 90444	*P. californicus*	11/13/98	BCN	>5,120	—
TK 90779	*P. californicus*	06/11/98	EC1	>5,120	AV A0060207
TK 90586	*P. californicus*	06/11/98	EC2	>5,120	AV A0060211
TK 90599	*P. californicus*	06/11/98	EC2	>5,120	AV A0060209
TK 90785	*P. californicus*	06/11/98	EC2	>5,120	AV A0060210
TK 90496	*P. californicus*	06/11/98	EC2	320	—

^a^BCN, Bear Canyon Trailhead; EC1, El Cariso #1; EC2, El Cariso #2. ^b^The antibody titer was the reciprocal of the highest dilution of the blood sample for which the AOD was >0.200 (see text). —, No viral isolate.

The sequence of the 616-nt fragment of the NP gene of isolate A0070039 was compared with the sequence of the homologous region of the NP genes of WWAV strains AV 9310135, AV 98490013, AV 96010025, AV 96010151, AV 96010024, and AV A0400174, TAMV, the 13 South American arenaviruses, LCMV, and LASV. The GenBank database sequences included in the analyses were accession nos. U52180, AY012711, AY012720, AY012717, AY012710, AY012713, U43690, Y012687, U43687, U43689, K02734, U62561, U43685, U43686, U70802, X62616, U41071, M20304, U43688, U34248, M20869, and AF182272. The predicted amino acid sequences were aligned by using the computer program CLUSTAL W1.7 (17). The multiple nucleotide sequence alignment was constructed manually based on the results of the amino acid sequence alignment. The analyses of the multiple nucleotide sequence alignment were done by using programs in the computer software package PAUP*, version 4.0b8a (18). The maximum likelihood (ML) analysis used the GTR+I+G model of substitution and the heuristic search option with a gamma shape parameter and transition-transversion ratio (Ti/Tv) of 0.508 and 1.96, respectively. The Modeltest program (19) indicated that the GTR+I+G model of substitution best fit the data for the likelihood analysis. Genetic distances were calculated by using the p model and Kimura two-parameter distance model (20). Sequence identities were calculated by subtracting the uncorrected p model distances from 1.0. The neighbor-joining (NJ) analysis used the Kimura genetic distances. The maximum parsimony (MP) analyses (all characters weighted equally, first and second nucleotide positions only, and amino acid sequences predicted from nucleotide sequences) were restricted to informative characters, with nodal support estimated by bootstrap analysis (21). Bootstrap support for the results of the NJ and MP analyses was based on 1,000 pseudoreplicate data sets generated from the original multiple nucleotide sequence alignment.

## Results

Thirty-four rodents (2 dusky-footed woodrats, 5 brush mice, and 27 California mice) were captured and tested for antibody or infectious arenavirus. Antibody to an arenavirus was detected in 1 of 2 dusky-footed woodrats and 4 of 8 California mice captured at the Bear Canyon Trailhead, 1 of 11 California mice captured at El Cariso #1, and 4 of 8 California mice and none of 5 brush mice captured at El Cariso #2. Infectious arenavirus was isolated from 1 California mouse each captured at the Bear Canyon Trailhead and El Cariso #1, 3 California mice captured at El Cariso #2, and none of the 29 other rodents. The five virus-positive animals also were antibody-positive (Table).

Sequence identities among the 489-nt NP gene fragments generated from the five isolates from the California mice ranged from 96.3% to 100.0%, indicating that the isolates are strains of a single virus. When compared with other arenaviruses, the 616-nt NP gene fragment of isolate A0070039 exhibited the highest nucleotide sequence identity and predicted amino acid sequence identity with (in decreasing order) the NP gene of WWAV prototype strain AV 9310135 (72.7% and 82.9%, respectively), TAMV (72.6% and 76.6%, respectively), the 13 South American arenaviruses (ALLV, AMAV, FLEV, GTOV, JUNV, LATV, MACV, OLVV, PARV, PICV, PIRV, SABV, and TCRV: 55.5%-63.5% and 47.8%-62.0%, respectively), and LCMV and LASV viruses (51.5%-51.9% and 42.7%-43.4%, respectively). In the same analysis, nucleotide and amino acid sequence identities between WWAV and TAMV were 73.1% and 80.0%, respectively, and nucleotide and amino acid sequence identities among the 13 South American arenaviruses ranged from 54.9% to 76.5% and 47.3% to 86.2%, respectively. Collectively, these results indicate that the arenavirus isolated from the California mice is distinct from all other Tacaribe complex viruses. The name “Bear Canyon” is proposed to denote the geographic origin of the first isolate of this novel virus, and strain A0070039 is designated as the prototype strain of BCNV.

The results of the ML analysis of nucleotide sequence data (Figure), NJ analyses of Kimura two-parameter genetic distances, and MP analyses of nucleotide and amino acid sequence data all indicated that BCNV, WWAV, and TAMV are monophyletic and phylogenetically distinct from the South American arenaviruses, LCMV, and LASV. Bootstrap support for monophyly of BCNV, WWAV, and TAMV was 100% in both the NJ and MP analyses.

**Figure:**
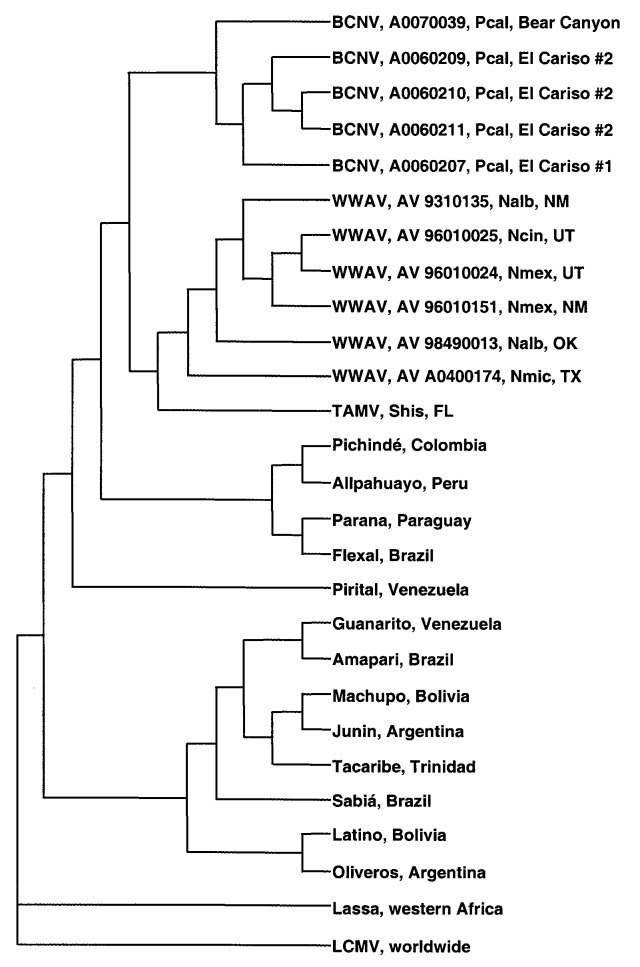
Phylogenetic relationships among Bear Canyon virus (BCNV) prototype strain A0070039, 4 other BCNV isolates from California mice (*Peromyscus californicus*), Whitewater Arroyo virus (WWAV), Tamiami virus (TAMV), and 15 other arenaviruses, based on a maximum likelihood analysis of a fragment of the nucleocapsid protein gene. The string of characters following “BCNV” or “WWAV” denotes the virus strain. LCMV, lymphocytic choriomeningitis; Pcal, *Peromyscus californicus*; Nalb, *Neotoma albigula*; Ncin, *N. cinerea*; Nmex, *N. mexicana*; Nmic, *N. micropus*; Shis, *Sigmodon hispidus*; NM, New Mexico; UT, Utah; OK, Oklahoma; TX, Texas; FL, Florida.

The ML analysis of nucleotide sequence data and MP analysis of amino acid sequence data placed WWAV in a sister relationship to TAMV and the WWAV–TAMV lineage in a sister relationship to the BCN lineage. However, bootstrap support for the WWAV–TAMV lineage in the MP analysis was low, i.e., 51%. The MP analysis of all nucleotide positions placed BCNV in a sister relationship to WWAV and the BCNV–WWAV lineage in a sister relationship to the TAMV lineage. However, bootstrap support for the BCN–WWAV lineage in that analysis was only 53%. The NJ analyses of nucleotide sequence data (with and without third position bases) indicated that BCNV, WWAV, and TAMV represent a trichotomy in the evolution of the Tacaribe complex viruses. Similarly, the MP analysis of nucleotide sequence data with third position bases excluded indicated that BCNV, WWAV, and TAMV represent a trichotomy in the evolution of the Tacaribe complex viruses. Sequence data representative of a larger fragment of the NP gene or a different region of the arenavirus genome may enable resolution of the phylogenetic relationships among the three North American viruses. Alternatively, the BCNV–WWAV–TAMV trichotomy may be the result of an essentially simultaneous divergence from the last common ancestor of the three North American arenaviruses. In that scenario, it would be difficult, if not impossible, to elucidate relationships among viruses in the North American (BCNV–WWAV–TAMV) lineage.

## Discussion

Previous studies of wild rodents in coastal southern California (Los Angeles, Orange, San Bernardino, San Diego, and Ventura Counties) showed antibody to an arenavirus in dusky-footed and desert woodrats, a brush mouse, California mice, a cactus mouse, deer mice, and western harvest mice (3,9). The isolation of BCNV from California mice is the first unequivocal evidence that a Tacaribe complex virus occurs in California and that the virus family *Arenaviridae* is naturally associated with the rodent genus *Peromyscus*. Further work is needed to determine the identity of the Tacaribe complex virus(-es) associated with woodrats, brush mice, cactus mice, deer mice, and western harvest mice in southern California.

In a recent study (5), WWAV was isolated from white-throated woodrats captured in northwestern New Mexico and western Oklahoma, a bushy-tailed woodrat (*N. cinerea*) in southern Utah, Mexican woodrats (*N. mexicana*) in central New Mexico and southern Utah, and southern plains woodrats (*N. micropus*) in southern Texas. The broad geographic association of WWAV with the rodent genus *Neotoma* suggests that WWAV is the arenavirus associated with dusky-footed woodrats in the Santa Ana Mountains.

The high prevalence of infection (50%) in California mice at El Cariso #2 and isolation of BCNV from California mice captured at the Bear Canyon Trailhead and El Cariso #1 indicates that the California mouse is the principal host of BCNV. However, the infections in the California mice could be the result of horizontal virus transmission from dusky-footed woodrats or another rodent that was not well represented in our study.

The geographic range of *P. californicus* extends from central California to San Quintin in Baja California (22). Throughout that range, the California mouse oftentimes is closely associated with middens of dusky-footed woodrats (22). Thus, the antibody-positive woodrat captured at the Bear Canyon Trailhead may have been infected with BCNV as a result of contact with infected California mice. Conversely, the California mice may have been infected with the virus as a result of contact with infected dusky-footed woodrats.

Human disease caused by Tacaribe complex viruses has been studied almost exclusively in South America. The results of our study indicate that there are substantial genetic differences among BCNV, WWAV, and TAMV. The genetic sequence differences and similarities among these viruses should be considered in the development of molecular-based assays for diagnosis of human disease caused by North American arenaviruses.

## References

[1] Buchmeier MJ, Bowen MD, Peters CJ. *Arenaviridae*: The viruses and their replication. In: Knipe DM, Howley PM, Griffin DE, Lamb RA, Martin MA, Roizman B, et al., editors. Fields virology. 4th ed. Philadelphia: Lippincott, Williams, and Wilkins; 2001, p. 1635–68.

[2] Childs JE, Peters CJ. Ecology and epidemiology of arenaviruses and their hosts. In: Salvato MS, editor. The Arenaviridae. New York: Plenum Press; 1993, p. 331–84.

[3] Kosoy MY, Elliot LH, Ksiazek TG, Fulhorst CF, Rollin PE, Childs JE, Prevalence of antibodies to arenaviruses in rodents from the southern and western United States: evidence for an arenavirus associated with the genus *Neotoma.* Am J Trop Med Hyg. 1996;54:570–5. 8686773 10.4269/ajtmh.1996.54.570

[4] Fulhorst CF, Bowen MD, Ksiazek TG, Rollin PE, Nichol ST, Kosoy MY, Isolation and characterization of Whitewater Arroyo virus, a novel North American arenavirus. Virology. 1996;224:114–20. 10.1006/viro.1996.0512 8862405

[5] Fulhorst CF, Charrel RN, Weaver SC, Ksiazek TG, Bradley RD, Milazzo ML, Geographic distribution and genetic diversity of Whitewater Arroyo virus in the southwestern United States. Emerg Infect Dis. 2001;7:403–7. 11384516 10.3201/eid0703.010306 PMC2631812

[6] Berge T, ed. International catalogue of arboviruses and certain other viruses of the world (supplement). Tamiami (TAM), strain W-10777. Am J Trop Med Hyg. 1970;19:1157–8.4395318

[7] Calisher CH, Tzianabos T, Lord RD, Coleman PH. Tamiami virus, a new member of the Tacaribe group. Am J Trop Med Hyg. 1970;19:520–6. 5446318 10.4269/ajtmh.1970.19.520

[8] Jennings WL, Lewis AL, Sather GE, Pierce LV, Bond JO. Tamiami virus in the Tampa Bay area. Am J Trop Med Hyg. 1970;19:527–36. 5446319 10.4269/ajtmh.1970.19.527

[9] Bennett SG, Milazzo ML, Webb JP Jr, Fulhorst CF. Arenavirus antibody in rodents indigenous to coastal southern California. Am J Trop Med Hyg. 2000;62:626–30. 11289675 10.4269/ajtmh.2000.62.626

[10] Fulhorst CF, Milazzo ML, Bradley RD, Peppers LL. Experimental infection of *Neotoma albigula* (Muridae) with Whitewater Arroyo virus (*Arenaviridae*). Am J Trop Med Hyg. 2001;65:147–1. 11508391 10.4269/ajtmh.2001.65.147

[11] Bowen MD, Peters CJ, Nichol ST. The phylogeny of New World (Tacaribe complex) arenaviruses. Virology. 1996;219:285–90. 10.1006/viro.1996.0248 8623541

[12] Bowen MD, Peters CJ, Nichol ST. Phylogenetic analysis of the *Arenaviridae*: patterns of virus evolution and evidence for cospeciation between arenaviruses and their rodent hosts. Mol Phylogenet Evol. 1997;8:301–16. 10.1006/mpev.1997.0436 9417890

[13] Bowen MD, Rollin PE, Ksiazek TG, Hustad HL, Bausch DG, Demby AH, Genetic diversity among Lassa virus strains. J Virol. 2000;74:6992–004. 10.1128/JVI.74.15.6992-7004.2000 10888638 PMC112216

[14] Weaver SC, Salas RA, de Manzione N, Fulhorst CF, Duno G, Utrera A, Guanarito virus (*Arenaviridae*) isolates from endemic and outlying localities in Venezuela: sequence comparisons among and within strains isolated from Venezuelan hemorrhagic fever patients and rodents. Virology. 2000;266:189–95. 10.1006/viro.1999.0067 10612673

[15] Weaver SC, Salas RA, de Manzione N, Fulhorst CF, Travasos da Rosa AP, Duno G, Extreme genetic diversity among Pirital virus (*Arenaviridae*) isolates from western Venezuela. Virology. 2001;285:110–8. 10.1006/viro.2001.0954 11414811

[16] Gonzalez JP, Sanchez A, Ricco-Hesse R. Molecular phylogeny of Guanarito virus, an emerging arenavirus affecting humans. Am J Trop Med Hyg. 1995;53:1–6. 7542842

[17] Thompson JD, Higgins DG, Gibson TJ. CLUSTAL W: improving the sensitivity of progressive multiple sequence alignment through sequence weighting, position-specific gap penalties and weight matrix choices. Nucleic Acids Res. 1994;22:4673–80. 10.1093/nar/22.22.4673 7984417 PMC308517

[18] Swofford DL. PAUP*. Phylogenetic analysis using Parsimony (*and other methods), version 4.0b8a. Sunderland (MA): Sinauer Associates; 2001.

[19] Posada D, Crandall KA. Modeltest: testing the model of DNA substitution. Bioinformatics. 1998;14:817–8. 10.1093/bioinformatics/14.9.817 9918953

[20] Kimura M. A simple method for estimating evolutionary rate of base substitutions through comparative studies of nucleotide sequences. J Mol Evol. 1980;16:111–20. 10.1007/BF01731581 7463489

[21] Felsentein J. Confidence limits on phylogenies: an approach using the bootstrap. Evolution Int J Org Evolution. 1985;39:783–91. 10.2307/2408678 28561359

[22] Merritt JF. *Peromyscus californicus*. In: Anderson S, editor. Mammalian species. No. 85. Old Dominion University, Norfolk (VA): The American Society of Mammalogists; 1978. p. 1–6.

